# Application of Arbuscular Mycorrhizal Fungi in Vineyards: Water and Biotic Stress Under a Climate Change Scenario: New Challenge for Chilean Grapevine Crop

**DOI:** 10.3389/fmicb.2022.826571

**Published:** 2022-03-03

**Authors:** Paula Aguilera, Nancy Ortiz, Ninozhka Becerra, Alessandra Turrini, Felipe Gaínza-Cortés, Patricia Silva-Flores, Ana Aguilar-Paredes, Juan Karlo Romero, Emilio Jorquera-Fontena, María de La Luz Mora, Fernando Borie

**Affiliations:** ^1^Scientific and Technological Bioresource Nucleus, Universidad de La Frontera, Temuco, Chile; ^2^Department of Agriculture, Food and Environment, University of Pisa, Pisa, Italy; ^3^Center for Research and Innovation – Viña Concha y Toro, Pencahue, Chile; ^4^Centro de Investigación de Estudios Avanzados del Maule, Vicerrectoría de Investigación y Postgrado, Talca, Chile; ^5^Centro del Secano, Facultad de Ciencias Agrarias y Forestales, Universidad Católica del Maule, Talca, Chile; ^6^Programa de Restauración Biológica de Suelos, Centro Regional de Investigación e Innovación para la Sostenibilidad de la Agricultura y los Territorios Rurales (CERES), Quillota, Chile; ^7^Vicerrectoría de Investigación y Estudios Avanzados, Pontificia Universidad Católica de Valparaíso, Valparaíso, Chile; ^8^Departamento de Ciencias Agropecuarias y Acuícolas, Universidad Católica de Temuco, Temuco, Chile; ^9^Facultad de Recursos Naturales, Universidad Católica de Temuco, Temuco, Chile

**Keywords:** grapevine crops, mycorrhiza, biotic stress, water stress, AMF inoculation

## Abstract

The crop *Vitis vinifera* (L.) is of great economic importance as Chile is one of the main wine-producing countries, reaching a vineyard area of 145,000 ha. This vine crop is usually very sensitive to local condition changes and agronomic practices; therefore, strategies to counteract the expected future decrease in water level for agricultural irrigation, temperature increase, extreme water stress (abiotic stress), as well as increase in pathogenic diseases (biotic stress) related to climate change will be of vital importance for this crop. Studies carried out in recent years have suggested that arbuscular mycorrhizal fungi (AMF) can provide key ecosystem services to host plants, such as water uptake implementation and enhanced absorption of nutrients such as P and N, which are key factors for improving the nutritional status of the vine. AMF use in viticulture will contribute also to sustainable agronomic management and bioprotection against pathogens. Here we will present (1) the current status of grapevines in Chile, (2) the main problems in grapevines related to water stress and associated with climate change, (3) the importance of AMF to face water stress and pathogens, and (4) the application of AMF as a biotechnological and sustainable tool in vineyards.

## Introduction

*Vitis vinifera* (L.) is a crop of economic importance, whose value depends largely on the quality of the fruit (Holland et al., 2014). The total vineyard planted area for the year 2020 was 7.3 million hectares, while global wine production was at 260 million hectoliters [International Organisation of Vine and Wine [OIV], 2021a]. During a conference at the OIV headquarters in Paris, the first estimates of world wine production during the year 2021 were presented. The projection was historically low, but it was a very positive year for the vineyards of the Southern Hemisphere where the climatic conditions were relatively favorable for wine production, such as in South American countries [International Organisation of Vine and Wine [OIV], 2021b]. However, the increase in greenhouse gases is already generating changes at the biogeochemical levels, causing temperature rises and an increase in CO_2_ concentration levels interacting with water deficit. Moreover, it is estimated that the global temperature for the year 2100 will increase between 2.2 ± 0.5 and 3.7 ± 0.7°C. Similarly, atmospheric CO_2_ would present new values at 669.7 and 935.9 ppm (IPCC, 2013). Because of this, the future abiotic stress (decrease in water level for agricultural irrigation, temperature increase, extreme water stress) as well as the increase in biotic stress (pathogenic diseases) related to climate change endangers the viability of the vine (*Vitis vinifera* L.). The use of biostimulants has been proposed to mitigate stresses associated with climate change (Del Buono, 2021) because inoculation of arbuscular mycorrhizal fungi (AMF) in vineyard soils can help this crop survive against abiotic stress (Massa et al., 2020; Nogales et al., 2021) and biotic stress (Hao et al., 2012; Bruisson et al., 2016). Although this literature review is not entirely focused on the relationship between AMF and the Chilean grapevine crop, the objective is to generate a precedent that allows the study of these biotechnological tools as possible solutions to the main problems caused by climate change in Chilean vineyards.

### Vine Crops in Chile

Chile is an important wine-producing country, with a vineyard area of 145,000 ha (Servicio Agrícola Ganadero [SAG], 2020). The vine crop extends from Atacama to La Araucanía regions, concentrating mainly in Central Chile between the O’Higgins and Maule regions (≈100,000 ha). The variety with the widest planting area is Cabernet Sauvignon, which reaches a proportion of 40% with 40,053 ha planted (Servicio Agrícola Ganadero [SAG], 2020). Chilean viticulture has stood out in exports, with Chile being the fourth wine exporter in 2018 (Varas et al., 2021), thanks to the origin denomination, grape cultivars, organoleptic characteristics, and sustainable practices (ProChile, 2018). However, as a result of the COVID-19 crisis in 2020, the resulting economic impact affected the wine sector, as international wine trade presented a reduction in value between 6.3 and 10.7% compared to 2019—a product of the fall in imports, consumption, and the difficulties to finish the harvests in the southern hemisphere [International Organisation of Vine and Wine [OIV], 2020]. However, in 2021, the total export of wine from Chile increased by 11.7% and that of wines with designation of origin increased by 13.8% compared with 2020, respectively.

In the recent years, there has been a growing consumption of food as well as healthy and better-quality beverages in developed countries (Lee and Yun, 2015), for which organic agricultural activities have acquired great importance worldwide. Consequently, the Chilean wine industry has developed a sustainability code to incorporate sustainable practices in companies of different sizes, in which certified companies can bear the “Certified Sustainable Wine of Chile” seal. In addition to projects within the framework of the R + D consortium to improve sustainability with respect to climate change, human impact, and wine-growing areas (Acuña, 2015), it is important to move toward organic viticulture that seeks to protect local biodiversity and establish organic vine crops. Chile is known worldwide in the food industry as a reliable supplier of healthy and safe food which is highly valued in international markets (ProChile, 2018).

## Main Problems in Chilean Grapevines

The crop of vines is usually very sensitive to local condition changes and the agronomic practices used (Massa et al., 2020); therefore, problems are generated mainly as a consequence of climate change. Added to the above-mentioned factor is the decrease in surface and groundwater water level, leading to less water available for agricultural needs (Pavithra and Yapa, 2018). Climate change will also affect biotic stresses, with an increase in the incidence of pathogens such as fungi (Díaz et al., 2013; Latorre et al., 2015; Gaínza-Cortés et al., 2020; Lolas et al., 2020), nematodes (Aballay et al., 2001), and even viruses (Fiore et al., 2008) that will strongly affect the vines and their fruits.

### Climate Change and Water Stress in Chilean Vineyards

A vine is a plant that has thick woody roots, which grows at an optimal temperature ranging between 25 and 30°C (Arribillaga and Reyes, 2021). This plant has low water requirements compared to other species of herbaceous-woody fruit crops. It is estimated that a vine needs between 280 and 300 L of water to form 1 kg of dry matter; however, it has the ability to adapt to mild conditions of water stress, thanks to osmotic adjustment at the cellular level (Leiva et al., 2017). When grown under these conditions, vine plants tend to increase the beneficial primary and secondary metabolites, thus increasing the production of quality wines (Griesser et al., 2015) since they are directly related to the organoleptic properties of the berries and, therefore, the wine.

Because increases in air CO_2_ concentration lead to increases in global temperature, grapevine responses must be evaluated considering both factors. Through free air CO_2_ enrichment experiments, grapevine biomass, photosynthesis, and fruit production normally increase (Bindi et al., 2001; Moutinho-Pereira et al., 2009), with no effect on fruit and wine quality (Bindi et al., 2001; Gonçalves et al., 2009; Arrizabalaga-Arriazu et al., 2020b; Wohlfahrt et al., 2021). In contrast, the individual effect of elevated CO_2_ stimulating grapevine production was reduced or completely canceled when elevated CO_2_ was combined with elevated temperature (Bindi et al., 2001). Under similar conditions, berry ripening, sugar accumulation, and malic acid breakdown were hastened in grapevine cv. Tempranillo (Arrizabalaga-Arriazu et al., 2020a). The effect of climate change-related factors, elevated CO_2_, and elevated temperature interacting with water deficit has received less attention in the literature. According to Salazar-Parra et al. (2010), the combined action of these factors significantly impacts berry quality traits; however, Kizildeniz et al. (2015) reported a high genotype dependency response across the combined factors. In grapevine cv. Tempranillo, Salazar-Parra et al. (2010) showed that increased CO_2_ concentration (700 μmol CO_2_ mol^–1^ air), elevated temperature (28/18 vs. 24/14°C, day/night), and partial irrigation (at 40% of field capacity) decreased the berry malic acid and total anthocyanin concentrations, while anthocyanin extractability was facilitated.

Though it has been shown that the effects sought with deficit irrigation are attenuated by very high temperatures (Torres et al., 2018a), in Chile most of the vineyard areas are subjected to seasonal water deficits (during spring and summer), especially in vineyards in the north of the country where a semi-arid climate is dominant (Bavestrello-Riquelme et al., 2012). Although protocols have been established for the optimization of water use in these areas, such as water reuse or regulated deficit irrigation, which allow the physiological stress of vines to overcome [International Organisation of Vine and Wine [OIV], 2016], the low levels of water available and the long periods of drought cause extreme water stress in grapevines. Such stress interferes with plant development by limiting the absorption of water and nutrients (Sardans et al., 2008) and altering fundamental biochemical and physiological processes such as photosynthesis and respiration, among others (Palliotti et al., 2014). Cell damage can also be generated by the formation of reactive oxygen species.

Currently, the decrease in water level is beginning to be noticed in Chile. In total, 95% of the vineyards have problems with water supply. It is estimated that rainfall in the Maipo Valley will decrease by approximately 20% by 2050 (Fuenzalida et al., 2007). This will lead to an increase of 3–4°C in the average temperature in the area, affecting river discharges and seasonality [Comisión Económica para América Latina y el Caribe [CEPAL], 2012]. In addition, it is estimated that, in Mediterranean climates, the surface area destined to viticulture will decrease between 25 and 73%, while for Chile the vine-growing area will decrease by 47% (Hannah et al., 2013). Considering that most of the premium wine-producing valleys in Chile (Maipo, Cachapoal, and Colchagua) will be mostly inadequate and that the suitability of other regions (Aconcagua and Maule) will decrease considerably, being affected by the decrease in the discharge of water (20–30% by 2050), this will lead to a possible decrease in the use of water to cool the grapes and the increased need for irrigation (Hannah et al., 2013). Many viticultural areas in Chile have been facing water shortages big enough to threaten the economic viability of the industry. Interestingly, wine production areas have been moving fast toward the south to areas with higher precipitation but with lower temperatures (Jorquera-Fontena and Orrego-Verdugo, 2010). As a result, the southern regions have experienced an increase in planted areas, mostly with cool climate varieties such as Chardonnay and Pinot noir [Servicio Agrícola Ganadero [SAG], 2018]. Since the 19th century, based on productive quality and organoleptic and enological potential, Chile has focused its wine production on those traditional varieties of French origin, highlighting Cabernet Sauvignon for its adaptability to soil and climatic conditions and cultivation. The origin of the vine in Chile dates back to the 16th century, most likely with the arrival of the first Spanish colonizers to Chile from regions such as Andalusia and Extremadura (Cortés, 2005). In this way, Chile does not have indigenous varieties, but old Spanish varieties were rather adapted during the following centuries. These adapted varieties receive the traditional name of cultivar ‘País,’ with a wide genetic diversity that has aroused interest in the last decade in search for varieties adapted to stress conditions with an endemic genetic character (Lacoste, 2004; Gutiérrez-Gamboa et al., 2020a). However, these old varieties do not present an adequate enological potential. Today’s efforts are focusing on the use of rootstocks that can cope with the challenges of climate change. In this sense, there are works to recover heritage varieties in order to use them as rootstocks (Milla-Tapia et al., 2013; Franck et al., 2019). As a future trend is relevant to consider even though plantations have been slowly increasing in the southern regions, viticulture faces new limitations such as the production on Andisols, which are very acidic, phosphorus-fixing, and prone to induce aluminum (Al) toxicity (Aguilera et al., 2017, 2021a,b).

Therefore, long periods of drought will interfere negatively with wine production. Since the decrease in water triggers a series of effects, such as a decrease in nutrient uptake, the absorption of water and nutrients are closely related to their flow from the roots to the shoots of vine plants (Keller, 2005). Plant growth stagnation will follow because the roots must extend to absorb all the water lost in transpiration so as to generate cell expansion and maintain root and shoot growth and development (Hsiao and Xu, 2000). In the shoots, it implies the detachment of the leaves and the cessation of secondary growth (Lovisolo et al., 2010). A restriction of cell division and a stagnation or reduction in the size of the berries also occur during bunch growth (Ojeda et al., 2002; Egunez-Zalakain, 2016). In general, in soils with a low percentage of humidity, the roots present greater vertical growth in order to increase their possibility of water absorption (Tomaz et al., 2015). Along with the development and thickening of the roots, there are also increases of the xylem ring and cavity decreases in resistance to water deficiency, as water availability in early development influences the structure of the xylem (Munitz et al., 2018).

Under drought stress, the water potential of the xylem decreases, limiting the amount of water and nutrients that can be used in plant growth (Valentine et al., 2006)—thus generating a reduction of transpiration rate and permeability of cell membranes and altering active transport. Water stress influences many plant metabolic and enzymatic processes, such as nitrate reductase activity (Kohler et al., 2008) and soil enzymatic activities (Sardans et al., 2008). Photosynthesis is reduced as a result of stomatal closure (Goicoechea et al., 2005), which is functional to the maintenance of low water potential and in preventing dehydration (Griesser et al., 2015). Respiration, due to limited gas exchange, decreases the production of glycolysis key enzymes (Król and Weidner, 2017), nutrient metabolism, and growth regulators, thus directly affecting plant growth and nutrition (Kahil et al., 2015). Consequently, the growing demand for water resources will put Chile’s freshwater ecosystems at risk [Ministerio de Medioambiente [MMA], 2011; Comisión Económica para América Latina y el Caribe [CEPAL], 2012; Hannah et al., 2013]. It is known that there is a positive relationship between altitude, air temperature, and the duration of the annual cycle of the vine (Falcão et al., 2010; Gutiérrez-Gamboa et al., 2020b). However, there are few studies that reveal this relationship with respect to the conditions of Chile in the main wine valleys and likewise with representative studies associated with altitude in relation to coastal influence (Coastal Range) (Gutiérrez-Gamboa and Moreno-Simunovic, 2019). As a consequence, the vineyards will move further south, establishing themselves in areas such as Valdivia or Chiloé (Bascopé, 2013) or changing to a productive system that is more resilient to climate change.

### Climate Change and Biological Pathogens of Chilean Grapevines

Chilean grapevines are currently affected by several pathogens such as fungi, nematodes, and viruses, which generate the greatest production losses in viticulture—mainly in plant growth and mortality. Among the most important pathogens, it is possible to find fungi that considerably affect vineyard production, berry quality, and plant longevity. In the first place, there is a group of fungi that produces what is generally known as grape trunk diseases or vine wood disease, which consists of a great diversity of pathogenic fungi that attack the woody part of the vine (trunk), causing the same symptoms such as necrosis, delayed development, chlorotic leaves, and plant death. Within the most common pathogenic fungi are *Phaeomoniella chlamydospora*, *Diplodia seriata*, and *Inocutis* sp. (Díaz et al., 2013) as well as the recently reported and widely distributed *Eutypa lata*, an important pathogen in syndromes caused by wood diseases (Gaínza-Cortés et al., 2020; Lolas et al., 2020) and which is considered among the most destructive pathogens generating the greatest economic loss in grapevines. The low control over such diseases is caused by the difficulty in the identification of the infective pathogen/s (Fontaine et al., 2016; Gaínza-Cortés et al., 2020). Another pathology of Chilean grapevines is the epidemics of gray mold, which cause considerable losses in fruit yield and quality worldwide (Latorre et al., 2015). In Chile, the rot is caused by *Cladosporium* spp., which is very common in grape varieties for the production of red wine, particularly in Cabernet Sauvignon (Briceño and Latorre, 2008).

Additionally, in the central area of Chile, a large part of the vineyards shows infestation by nematodes, which represent another aspect of the serious problems affecting Chilean wine production. Nematodes cause damage to the roots, which allows the entry of other phytopathogens such as bacteria, viruses, and fungi (Aballay et al., 2001). In addition, they generate loss of vigor and quality, even reaching to the total loss of plants, especially in sensitive varieties (Aballay et al., 2009). A study carried out by Fiore et al. (2008) revealed that Chilean grapevines showed a highly variable percentage of virus infection, with ranges varying between 21 and 74% and with a more common presence of seven viruses in the following order: grapevine fan leaf virus (GFLV), grapevine leafroll-associated virus (GLRaV-1, −2, and −3), grapevine vitiGVA, GFkV, and GRSPaV, which also cause a decrease in the production of grapevines but in a lower proportion compared with the pathogens mentioned previously.

In the context of climate change, the increasing temperatures as well as the decrease in precipitation are predicted to modify the distribution of the pathogens as well as the incidence, severity of infection, and even the outbreak time (Salinari et al., 2006; Bove et al., 2020). In relation to the high concentration of CO_2_ generated in the atmosphere, it could affect the response of the transcriptional system in grapevines. In this sense, differences have been found in the expression level of a subset of herbivory-responsive genes when an RT-qPCR evaluation was performed (Reineke and Selim, 2019). Since this can occur, it is highly likely that this could have detrimental effects on *V. vinifera* crops subjected to diseases by fungi, nematodes, and viruses.

## Importance of Arbuscular Mycorrhizal Fungi

It is estimated that arbuscular mycorrhizal fungi (AMF) form a symbiotic association with 78% of vascular plants, representing the most common mycorrhizal symbiosis on earth (Brundrett and Tedersoo, 2018). Specifically, arbuscular mycorrhiza (AM) occurs when fungal hyphae recognize signal molecules, such as hormones, flavonoids, or derivatives of volatile organic compounds released through the radical exudates at the rhizosphere level (Saparrat et al., 2020). In particular, a group of phytohormones, called strigolactones, is important for the elicitation of differential morphogenesis represented by extensive hyphal branching (Giovannetti et al., 1993; Akiyama et al., 2005) close to the roots, which is essential to reach the roots and form appressoria, the first step of AMF colonization. After appressoria differentiation, AMF colonize the roots, forming intracellular and intercellular hyphae, vesicles, and arbuscules. Arbuscules are the structures where a bidirectional exchange of soluble compounds is generated and where mineral nutrients are transferred from the fungus to the plant while carbon compounds are delivered from the plant to the fungus, contributing also to global warming mitigation (Pischl and Barber, 2017). Studies indicating that AMF communities are affected by the identity of the host plant have been conducted (Torrecillas et al., 2012; Holland et al., 2014). Holland et al. (2014) found more than 40 different AMF taxa to be associated with both grapevine and interrow root, mainly highlighting the genera *Funneliformis* and *Rhizophagus*, but these communities differ depending on the identity of the host plant.

The level of extension and diversity of plants that AMF can colonize is widely known. However, these fungi are very sensitive to the changing environment. For this reason, they are also directly or indirectly affected by climate change interfering in colonization, distribution, and diversity levels (Chourasiya et al., 2021). Further studies relate increased CO_2_ to a significant increase in AMF colonization, which can be justified with increased plant nutrient requirement due to increased C fixation (Zhu et al., 2016). Decrease in water availability would not negatively compromise AMF symbiosis. Moreover, there is a proportional relationship between the water status of the plant and the fungus. A lower water status generates higher colonization (Torres et al., 2021).

Accordingly, scientists have proposed the use of AMF as biostimulants. But how do vines affected by abiotic or biotic stress benefit from AMF inoculation? As vine is cultivated worldwide, not only in Chile, many investigations evaluated the advantages generated by AMF in vine crops by studying their effects on pest control, including nematodes and weeds. The benefit of AMF in the contexts of water stress as well as other abiotic stresses was also evaluated.

### Arbuscular Mycorrhizae in Water Deficit of Grapevines

Recent studies proved that the application of AMF on vines improves tolerance to abiotic stress, enhancing sustainable crops facing unfavorable climatic conditions, such as high temperatures or long periods of drought (Gianinazzi et al., 2010), which was consistent with previous research carried out on other types of annual plants (e.g., Schreiner and Linderman, 2005). AMF generate certain controversies among researchers, mainly when talking about certain factors that negatively affect them. This is the case of increased temperatures because while some researchers relate this factor to an increase in mycorrhizal abundance, others claim that it causes a decrease in colonization levels (Torres et al., 2018b). Edaphoclimatic factors also influence the colonization levels. A high content of phosphorus (P), thus increasing soil acidity, calcium (Ca), potassium (K), and total nitrogen in the soil, leads to lower AMF colonization rates (Sagadin et al., 2018). It has been shown that reducing soil moisture stimulates mycorrhizal colonization, which implies a direct relationship between vine water availability and mycorrhizal fungi. This relationship has been demonstrated to enhance the plant’s tolerance to drought conditions (Schreiner and Linderman, 2005), suggesting that AMF represent a suitable tool for grapevines to cope with the effects of climate change. However, doubts remain in relation to the levels of mycorrhization to face increases in temperature accompanied by decreases in water availability (Torres et al., 2018a). In general, mycorrhization influences the plant at the hormonal, physiological, and therefore metabolic levels, increasing grape yield and improving water use efficiency. It also regulates the ecology and stability of the rhizosphere (Kadam et al., 2020). Consequently, it is expected that AMF are increasingly used in sustainable agriculture in order to contrast the deficiency in water availability and to increase soil fertility (Schreiner, 2005).

As to the morphology of vine roots, they are mainly thick and woody, which makes it difficult to absorb water in areas with water deficit, but AMF colonization allows the roots to cover a large absorption area due to the length and size of the extraradical hyphae, which facilitate access to the smallest soil pores (Trouvelot et al., 2015). It has been observed that AMF inoculation promotes the development of the vine root system, with longer roots and more secondary roots, and vine health, with a lower incidence of diseases than non-inoculated plants (Krishna et al., 2006). Moreover, hyphae can absorb and transport a greater quantity of water compared with that absorbed by roots (Hayman, 1983). Mycorrhizal vines present higher levels of water potential and stomatal conductance in the leaves than non-mycorrhizal vines under the same drought stress and show beneficial effects on the assimilation of CO_2_, improving and regulating the use of water and increasing the photosynthetic rate (Nikolaou N. et al., 2003). Moreover, while reducing the hydraulic and stomal resistance, the mycorrhizal vine remains more turgid than the non-inoculated ones which wilt faster (Nikolaou N. A. et al., 2003).

As a result of the improvements caused by AMF in the absorption and efficient use of water, these microorganisms could alleviate vine transplant shock and increase the survival rate (Aguilar, 2017), also thanks to the biochemical changes occurring in mycorrhizal plants, such as the increase in the levels of chlorophyll, phenols, proline, and enzymes (Krishna et al., 2005). Proline is an osmoregulatory amino acid responsible for protecting and stabilizing enzymes or cell membrane structures that are sensitive to any water level alteration. Accordingly, the increase in proline levels indicates the ability of AMF to regulate or induce osmotic adjustment in inoculated vine plants, while the increase in nitrate reductase, due to the increased phosphorus absorption (P), allows a higher nitrogen assimilation, thus improving vegetative growth (Krishna et al., 2006). In addition, AMF influence the secretion of certain phytohormones such as cytokinins, which are able to regulate plant growth and development, promote cell division and organogenesis, and delay cell senescence (Nikolaou N. A. et al., 2003).

### Arbuscular Mycorrhizal Fungi and the Main Biological Pathogens of Chilean Grapevines

Because the use of pesticides is harmful to the environment and human health, a number of chemicals have been withdrawn from the market. In addition, pathogens are increasingly resistant to the use of these products of chemical origin (Gianinazzi et al., 2010). For this reason, the use of bioproducts based on AMF has gained importance, as they are used as bioprotectors against pathogens. These biostimulants could contribute to agronomic management focused on sustainable agriculture since the use of AMF would allow production increase, enhancing the quality of the final product and reducing or eliminating pathogens without damaging the environment and harming human health. Therefore, several studies have been carried out in order to evaluate AMF’s ability to affect different pathogens, how they influence production, and how they are related to current crop management. Regarding crop management, it has been reported that conventional management styles that use chemical fertilizers and pesticides influence the percentage of colonization and diversity of AMF in vines as a result of the alteration of the soil pH or mycelium disruptions (Massa et al., 2020).

The rhizosphere has a set of environmental conditions for many microorganisms of different biological origin as well as different ecological roles, and in order to infect a root, pathogens must compete with the rest of the microbiota present in the soil for a space in the rhizosphere and for plant nutrients (Chapelle et al., 2016). In this scenario, AMF participates in this competition; an example is represented by the use of the AMF species *Rhizoglomus intraradices* (*Glomus intraradices*) to counteract the action of fungi causing black foot on grapevines. The mechanism of action is not available, but it has been suggested that AMF reduce the infection sites for the pathogens or generate an increase in the absorption of nutrients such as P, N, K+, Ca+, (Fattahi et al., 2021), Cu, Zn, and Fe (Trouvelot et al., 2015; Nogales et al., 2019), improving vine nutritional status and, therefore, its capacity to tolerate pathogen infections (Petit and Gubler, 2006).

**FIGURE 1:**
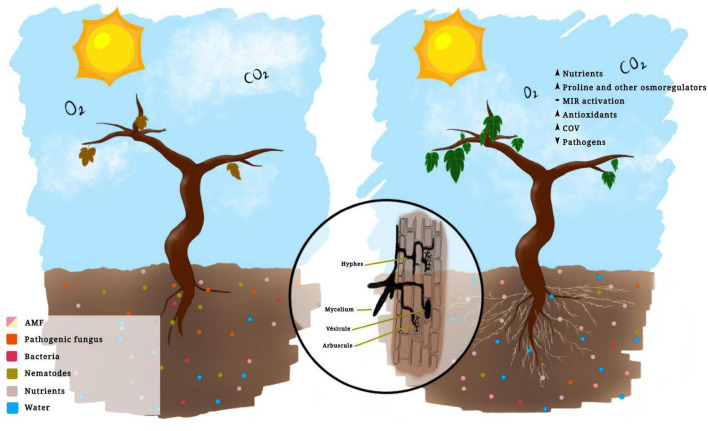
*Vitis vinifera* (L.) not inoculated (left) and inoculated with arbuscular mycorrhizal fungi (right).

The application of bioinoculants promotes greater plant growth in grapevines (Figure 1) by two mechanisms: directly, through greater absorption of nutrients and water through the hyphae, and indirectly, by influencing the synthesis and regulation of hormones and other compounds, such as volatile organic compounds (VOC) (Velásquez et al., 2020), whose main functions are to repel or attract predators for insects and nematodes that damage the plant (Maffei, 2010; Velásquez et al., 2020). As an example, the arbuscular mycorrhizal fungus *Funneliformis mosseae* increased plant growth and VOC concentration in grapevine plants (Velásquez et al., 2020). The ability to repel or tolerate pathogens will depend on the AMF species, although the repellent effect of AMF cannot be generalized, which is not applicable for the management of all pathogens (Pozo and Azcón-Aguilar, 2007). However, the application of AMF prior to infection by nematodes not only controls the population of these pathogens and the symptoms that they cause but also increases the average weight of fruits and plant growth (Aleandri et al., 2015).

It has been shown that plants develop a higher level of protection against pathogens when colonized by AMF, and this increase in resistance is known as mycorrhiza-induced resistance (MIR) (Pozo and Azcón-Aguilar, 2007; Jung et al., 2012; Trouvelot et al., 2015; Aguilar, 2017; Miozzi et al., 2019). MIR has been reported to be effective against soil pathogens such as insects that attack the roots and foliar fungi, generating local and systemic defense responses, i.e., although the symbiotic association originates in the roots, the effects are triggered throughout the plant (Kadam et al., 2020). MIR responses at the cellular level include the deposition of callose, a polysaccharide reinforcing plant cell walls against attackers, blocking the entry of pathogens and giving the plant additional time for the activation of subsequent defense mechanisms. Actually, plants colonized with AMF have a greater reserve of polysaccharides, thanks to the increase in photosynthesis rate (Sanmartín et al., 2020). The results of Nerva et al. (2021) suggest that AM symbiosis triggers MIR also in a model woody plant like the vine. MIR is mainly related to pathogens that affect the roots, such as fungi and nematodes, like *Xiphinema*, a vector nematode of the GFLV virus (Hao et al., 2012; Aguilar, 2017; Kadam et al., 2020). *R. intraradices* was used to control the levels of *Xiphinema* in grapevines as a result of active defense genes such as chitinase 1b, which interferes in the feeding and reproduction of nematodes (Hao et al., 2012). As to the management of the nematode *Meloidogyne incognita*, grapevines were inoculated with *Diversispora versiformis* (*Glomus versiforme*), which originated an increase in the transcription of the *VCH3* gene coding for chitinase, activating the defense responses against the nematode (Li et al., 2006).

Arbuscular mycorrhizal fungi promote and improve defenses already established by plants. One of the best-known mechanisms is the increased concentration of jasmonic acid (JA) in plant roots, which occurs when AMF infection begins in cortical cells because JA is a hormone that facilitates colonization and is closely related to induced systemic resistance (ISR) (Holland et al., 2019). When AMF initiate colonization, their microorganism-associated molecular patterns are recognized by the plant’s immune system that leads to the transient expression of colonization-activated immunity, generating signals by vascular tissues and inducing the preparation of the defenses, such as salicylic acid production and systemic acquired resistance (García-Garrido and Ocampo, 2002). In addition, AMF stimulate the production of abscisic acid, and radical exudates that send ISR signals to the root surface and hyphae are metabolized (Trouvelot et al., 2015).

Despite the benefits granted by these symbiotic fungi, the application of diverse AMF species and isolates should be justified and carefully studied before inoculation since some studies showed that AMF inoculation has increased fungal pathogen infections. Such data may be due to the synergistic effect of the AMF species used and the pathogen (Holland et al., 2019). However, in general, the application of AMF allows the suppression or reduction of pests and plant diseases by inducing systemic resistance (Jung et al., 2012).

## Arbuscular Mycorrhizal Fungi as Biotechnological Tools in Vineyards

Like most terrestrial plants, *V. vinifera* in field conditions is normally colonized by AMF. However, there are differences in the biodiversity of these microorganisms, depending on the properties of the soil (Balestrini et al., 2010) and the agricultural practices. More diverse and abundant AMF were observed in agroecological systems of Chilean vineyards as reported in Aguilar-Paredes et al. (unpublished data). It has been reported that, within Glomeromycota, the genus *Glomus* regularly occurred in vine crops (Likar et al., 2013). As a consequence, most investigations on the effects of AMF on vine crops used species of this genus as a source of inoculation (Table 1). Such studies showed that AMF are able to confer benefits to vines, including a higher *ex vitro* survival rate in micropropagated plants (Krishna et al., 2006) and greater tolerance to stress due to water deficit (Nikolaou N. et al., 2003; Nikolaou N. A. et al., 2003; Valentine et al., 2006). The AMF are multifunctional microorganisms that are in a dynamic equilibrium in the soil biome. With sustainable agriculture practices, we can promote all the benefits and develop vineyards resilient to a different stressor, including climate change (Aguilar-Paredes et al., 2020).

**TABLE 1:** Application and benefits of arbuscular mycorrhizal fungi in vineyards.

Species	AMF inoculation time	Evaluation time	Benefits/Effects	Stage/plant material	References
*Funneliformis mosseae* (*Glomus mosseae*)	Weeks before applying the normal irrigation and drought treatment. At the time of transplantation. Inoculum: pot medium + pieces of mycorrhizal corn roots	5 and 8 days	Greater foliar growth. Higher concentration of P in the leaves. Greater tolerance to water stress. Increase in water potential at dawn. Higher carbon assimilation rates	Cuttings seedlings Eight vine rootstocks	Nikolaou N. et al., 2003
*Funneliformis mosseae* (*Glomus mosseae*)	Weeks before applying the normal irrigation and drought treatment. At the time of transplantation. Inoculum: pot medium + pieces of mycorrhizal corn roots	– (The waiting time for evaluation does not appear)	Increased production of cytokinins Greater stomatal conductance Greater water potential in the leaves before sunrise	Grafted plants in pots Eight vine rootstocks	Nikolaou N. A. et al., 2003
*Rhizophagus manihotis* (*Glomus manihotis*) *Funneliformis mosseae* (*Glomus mosseae*) *Dentiscutata heterogama* (*Scutellospora heterógama*) *Acaulospora colombiana* (*Entrophospora colombiana*) *Acaulospora laevis* *Acaulospora scrobiculata* *Gigaspora gigantea*	At transplantation Inoculation: freshly collected rhizosphere + spores + hyphae + arbuscules and vesicles + root segments of mycorrhizal Rhodes grass	At transplantation 60 days after inoculation	Greater absorption of nutrients Increase in the concentration of proline Increased synthesis of chlorophyll, carotenoids and polyphenol oxidase	Seedlings derived from tissue culture, in the acclimatization stage Variety: Pusa Navrang	Krishna et al., 2005
*Scutellospora calospora* *Funneliformis mosseae* (*Glomus mosseae*) *Rhizoglomus intraradices* (*Glomus intraradices*)	Inoculum: AMF mix	8 and 16 weeks	Greater sprout growth	Plants in ultisol Variety: Pinot noir	Schreiner, 2005
*Rhizophagus manihotis* (*Glomus manihotis*) *Funneliformis mosseae* (*Glomus mosseae*) *Dentiscutata heterogama* (*Scutellospora heterógama*) *Acaulospora colombiana* (*Entrophospora colombiana*) *Acaulospora laevis* *Acaulospora scrobiculata* *Gigaspora gigantea*	At transplantation. Inoculum: freshly collected rhizosphere + spores + hyphae + arbuscules and vesicles + mycorrhizal Rhodes grass root segments	60 days after inoculation	Increased ex vitro survival rate of micropropagated seedlings Greater growth and development Higher photosynthetic rate Higher activity of nitrate reductase Increase in electron transport rate	Seedlings derived from hardening-stage tissue cultures Variety “Pusa Urvashi” from IARI	Krishna et al., 2006
*Diversispora versiformis* (*Glomus versiforme*)	At transplantation. 25 days prior to inoculation by the pathogen Inoculum: mixture of sand + spores, 8,000 units of potential inoculum (UPI)	1–13 after inoculation with the pathogen	VCH3 transcription increase	Mycorrhizal plants Variety: Cv. Shuangyou, susceptible to RKN *M. incognita*	Li et al., 2006
*Funneliformis mosseae* (*Glomus mosseae*)	Prior to drought stress. At transplantation Inoculum: clay + chlamydospores + mycorrhizal root fragments	4 weeks of drought	Higher biomass Increase in the concentration of proline Greater activity of the RuBisCO Increase in electron transport rate	1 year old plants Variety: Chenin blanc	Valentine et al., 2006
*Rhizoglomus intraradices* (*Glomus intraradices*)	Prior to infection with pathogens. At the time of sowing Inoculum: soil + 15 spores + roots of mycorrhizal Sudan grasses	8 months after inoculation with the pathogen	Counteracts the effects of blackfoot pathogens on grapevines	Callus cuttings *V. rupestris*	Petit and Gubler, 2006
*Rhizoglomus intraradices* (*Glomus intraradices*)	Before and after infection by the pathogen. At transplant and after Inoculum: spores + mycelium + soil + mycorrhizal onion root fragments	0 and 35 days after inoculation with the pathogen	Reduction of gall formation caused by Xiphinema Nematode population decline Higher fresh weight of the plants	Cuttings seedlings One rootstock	Hao et al., 2012
*Rhizoglomus intraradices* (*Glomus intraradices*)	Establishing autotrophic culture *in vitro* Inoculum: Commercial product called MYCOSYM from the Spanish company MYCOSYM-TRITON SL	13 weeks	Increase in the concentration of polyphenols	*In vitro* micropropagated seedlings Variety: Cabernet Sauvignon	Aguilar, 2017

Another aspect that is important to consider in using AMF as a biotechnological tool is to decide which AMF species should be considered as inoculum. The industry of AMF inoculum has grown at a high speed (Hart et al., 2018), and in Chile, most of the AMF fungal inoculum products are imported. As it is widely believed that AMF species are widespread, this can lead to the wrong idea that any species can be used anywhere (Rodriguez and Sanders, 2015). However, it has been shown that some AMF inoculants can reduce plant biodiversity and avoid the establishment of native plants (Middleton et al., 2015; Emam, 2016). Moreover, the origin of the inoculum was shown to be important in terms of using a biotechnological tool in a sustainable manner since a foreign inoculum, relative to a local one, could be detrimental to the native vegetation that might be surrounding a vineyard (Burkle and Belote, 2015). The preservation of the native surrounding vegetation or even vegetation corridors has recently been acknowledged as important in order to increase the habitat quality of agricultural systems since it provides valuable ecosystem services (Díaz-Forestier et al., 2021). However, there is still much research to be conducted in order to really assess the general benefits of using AMF inoculum (Hart et al., 2018). Consequently, to study the system of interest, in this case, vine plants–AMF–Chilean agricultural territories, is of paramount interest to truly achieve the desired benefits of AMF with vine plants in a climate change scenario.

## Conclusion

Given the key role that AMFs are likely to play with regard to alleviating abiotic and biotic stress in grapevine plants, it is important to conduct further research in laboratory experiments and in the field in the coming years. In fact, AMF inoculants will make a significant contribution to sustainable grapevine production systems in the face of climate change. In grapevine, only a few inoculants were used. Studies that focus on the identification of the diversity of AMF present in the grapevine in different edaphoclimatic conditions are required, and there may be dominant species that can effectively contribute to grapevine symbiosis in the grapevines. Upon recognizing areas most affected by climate change, through the knowledge of different species of AMF and the evaluation of their isolates, it will be possible to evaluate the effects of AMF selected in different stress conditions at the greenhouse level for their ability to alleviate both abiotic and biotic stresses in grapevine plants. Such isolates should be tested in subsequent field studies to assess the maintenance of their beneficial activities and could eventually be reproduced for use as biostimulants in more resistant and sustainable grapevine production systems. Finally and as mentioned at the beginning, although this bibliography does not establish the relationship between AMF and Chilean grapevine crop, it does allow us to extrapolate data and open the doors for research on this tool with our national variables.

## Author Contributions

PA, NO, NB, and AT contributed to the initial writing. FG-C and PS-F contributed from their experience from the agricultural and scientific sector. AA-P, JR, EJ-F, MM, and FB reviewed the writing. All authors contributed to the article and approved the submitted version.

## Conflict of Interest

The authors declare that the research was conducted in the absence of any commercial or financial relationships that could be construed as a potential conflict of interest.

## Publisher’s Note

All claims expressed in this article are solely those of the authors and do not necessarily represent those of their affiliated organizations, or those of the publisher, the editors and the reviewers. Any product that may be evaluated in this article, or claim that may be made by its manufacturer, is not guaranteed or endorsed by the publisher.
